# Syringomyelia as a complication of tuberculous meningoencephalitis

**DOI:** 10.11604/pamj.2016.25.141.10552

**Published:** 2016-11-11

**Authors:** Maha Ait berri, Abdelhadi Rouimi

**Affiliations:** 1 Department of Neurology, Military Hospital Moulay Ismail, Meknès, Morocco

**Keywords:** Syringomyelia, MR imaging, spinal tubercular arachnoiditis

## Image in medicine

Syringomyelia is a rare condition in which a cyst forms within your spinal cord, secondary syringomyelia following Tuberculousis is very rare. In the present paper, we report a case of tuberculous meningoencephalitis in a 30 year-old Morrocan male complicated six months after under anti-bacillary treatment by progressive quadriplegia due to granulomatous arachnoiditis. The spinal MRI showed an extensive complex syrinx within the cervical and thoracic cord same signal intensity as the cerebrospinal fluid. Arachnoiditis appeared to be the underlying mechanism in these late-onset cases. The availability of MRI has greatly improved our ability to both diagnose and follow these collections. Surgery is the only viable treatment for syringomyelia. The possibility of developing syringomyelia should be always considered in any patient with a history of central nervous system infection.

**Figure 1:**
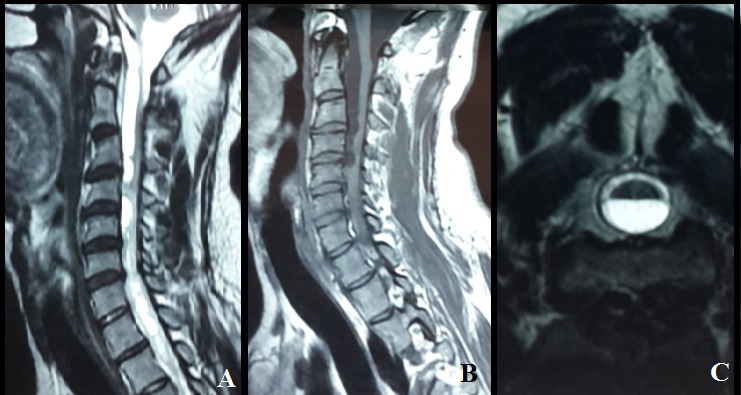
MRI of the neck shows syringomyelia cervical: A) sagittal T2; B) sagittal T1; C) axial T2

